# Sequencing of whole plastid genomes and nuclear ribosomal DNA of *Diospyros* species (Ebenaceae) endemic to New Caledonia: many species, little divergence

**DOI:** 10.1093/aob/mcw060

**Published:** 2016-04-20

**Authors:** Barbara Turner, Ovidiu Paun, Jérôme Munzinger, Mark W. Chase, Rosabelle Samuel

**Affiliations:** ^1^Department of Botany and Biodiversity Research, Faculty of Life Sciences, University of Vienna, Rennweg 14, 1030 Wien, Austria; ^2^IRD, UMR AMAP, TA A51/PS2, 34398 Montpellier Cedex 5, France; ^3^Jodrell Laboratory, Royal Botanic Gardens, Kew, Richmond, Surrey TW9 3DS, UK; ^4^School of Plant Biology, The University of Western Australia, Crawley, WA 6009, Australia

**Keywords:** *Diospyros*, genome skimming, island floras, New Caledonia, next-generation sequencing, nuclear ribosomal DNA, rapid radiation, complete plastid genomes

## Abstract

**Background and Aims** Some plant groups, especially on islands, have been shaped by strong ancestral bottlenecks and rapid, recent radiation of phenotypic characters. Single molecular markers are often not informative enough for phylogenetic reconstruction in such plant groups. Whole plastid genomes and nuclear ribosomal DNA (nrDNA) are viewed by many researchers as sources of information for phylogenetic reconstruction of groups in which expected levels of divergence in standard markers are low. Here we evaluate the usefulness of these data types to resolve phylogenetic relationships among closely related *Diospyros* species.

**Methods** Twenty-two closely related *Diospyros* species from New Caledonia were investigated using whole plastid genomes and nrDNA data from low-coverage next-generation sequencing (NGS). Phylogenetic trees were inferred using maximum parsimony, maximum likelihood and Bayesian inference on separate plastid and nrDNA and combined matrices.

**Key Results** The plastid and nrDNA sequences were, singly and together, unable to provide well supported phylogenetic relationships among the closely related New Caledonian *Diospyros* species. In the nrDNA, a 6-fold greater percentage of parsimony-informative characters compared with plastid DNA was found, but the total number of informative sites was greater for the much larger plastid DNA genomes. Combining the plastid and nuclear data improved resolution. Plastid results showed a trend towards geographical clustering of accessions rather than following taxonomic species.

**Conclusions** In plant groups in which multiple plastid markers are not sufficiently informative, an investigation at the level of the entire plastid genome may also not be sufficient for detailed phylogenetic reconstruction. Sequencing of complete plastid genomes and nrDNA repeats seems to clarify some relationships among the New Caledonian *Diospyros* species, but the higher percentage of parsimony-informative characters in nrDNA compared with plastid DNA did not help to resolve the phylogenetic tree because the total number of variable sites was much lower than in the entire plastid genome. The geographical clustering of the individuals against a background of overall low sequence divergence could indicate transfer of plastid genomes due to hybridization and introgression following secondary contact.

## INTRODUCTION

New Caledonia comprises an archipelago in the southern Pacific known for its characteristic, rich endemic flora (Lowry, 1998; Morat *et al.*, 2012). Due to its complex geological history, New Caledonia features a mosaic of soil types (Pelletier, 2006; Maurizot and Vendé-Leclerc, 2009), which, in combination with its elevational and climatic heterogeneity, results in many different habitats. One of the genera that has adapted to a wide range of these habitats is *Diospyros* (Ebenaceae).

*Diospyros* is a large genus of woody dioecious plants found worldwide in the tropics and subtropics, including 31 species in New Caledonia. Previous studies based on plastid markers (Duangjai *et al.*, 2009) showed that *Diospyros* colonized New Caledonia at least four times via long-distance dispersal. Two of the successful dispersal events each resulted in a single species that still persists; an additional dispersal event led to a small clade comprising five species; and yet another event gave rise to a putatively rapidly radiating group of 24 endemic species. These 24 species have been shown to be highly similar genetically using low-copy nuclear and plastid markers (Duangjai *et al.*, 2009; Turner *et al.*, 2013*a*). Most of these closely related species are morphologically and ecologically clearly differentiated, and current species delimitations (White, 1993) have been generally confirmed by analyses of amplified fragment length polymorphisms (AFLPs; Turner *et al*., 2013*b*) and restriction site-associated DNA sequencing (RADseq; Paun *et al.*, 2016).

On New Caledonia, *Diospyros* species are found in many habitats, but they often grow in proximity to each other. At some localities, several species are microsympatric, which allows interspecific gene flow if reproductive isolation is still incomplete. Dating analysis based on four plastid and two low-copy nuclear DNA regions showed that the ancestors of this group of New Caledonian *Diospyros* species arrived in New Caledonia around 9 million years ago (Turner *et al.*, 2013*a*). Given that *Diospyros* includes long-lived perennial plants, it becomes obvious that they have evolved relatively recently. Resolving the phylogenetic relationships in such a young group of rapidly radiating and potentially hybridizing taxa poses significant challenges (Glor, 2010). We test here the usefulness of next-generation sequencing (NGS)-based genome skimming to obtain phylogenetic data, in particular by sequencing whole plastomes and the full-length nuclear ribosomal DNA region (i.e. nrDNA).

The plastid genome has proved useful for molecular phylogenetic investigations of plants at different taxonomic levels. In the past two decades, sequences from the plastid genome have been extensively used to infer phylogenetic relationships among plants (e.g. Chase *et al.*, 1993; Barfuss *et al.*, 2005; Duangjai *et al.*, 2009; Russell *et al.*, 2010). Uniparental inheritance, low mutation rates and high copy number are well-known features of plastid genomes and the basis for their standard usage in plant systematics. Due to the slow rate of evolution of the plastid genome, the level of variation is often low compared with nuclear DNA in general and mitochondrial markers in animals (Schaal *et al.*, 1998). The high level of conservation has prevented the development of a universally applicable single barcoding region in plants (CBOL Plant Working Group, 2009). Recently, whole-plastid genome sequencing has become affordable, and this has been employed to generate more highly resolved phylogenetic trees (e.g. Ku *et al.*, 2013; Yang *et al.*, 2013; Barrett *et al.*, 2014; Malé *et al.*, 2014).

Genes coding for nuclear ribosomal RNA are found in the genome in multiple copies arranged in tandem repeats, and therefore it is feasible to obtain full-length sequences from low-coverage NGS approaches. Because of concerted evolution, these thousands of copies mostly behave as single-copy genes, and potential evidence of hybridization is normally eliminated within a few generations (Chase *et al.*, 2003). Each nrDNA repeat consists of coding and non-coding elements. In plants, the four rRNA genes are arranged in two clusters. One of these clusters comprises three genes (18S, 5·8S and 26S) separated by two internal transcribed spacers (ITSs), the external transcribed spacer (ETS) and the non-transcribed spacer (NTS). The second cluster includes the coding region of one rRNA (5S) and a spacer between the repeats. The nrDNA genes are relatively conserved but contain enough variation that they have been used for phylogenetic reconstructions at higher taxonomic levels (Maia *et al.*, 2014). The non-coding spacers, ITS and ETS, between the genes are much more variable and represent a useful source of phylogenetic information among closely related species (e.g. Devos *et al.*, 2006; Sanz *et al.*, 2008; Akhani *et al.*, 2013; Nürk *et al.*, 2013; Zhu *et al.*, 2013).

Here we aimed to use whole plastid genomes as well as complete sequences of the nrDNA for phylogenetic analyses for species of the New Caledonian *Diospyros* to investigate whether this approach would produce an improved estimate of phylogenetic relationships in this putatively rapidly radiating group.

## MATERIALS AND METHODS

**Fig. 1.**
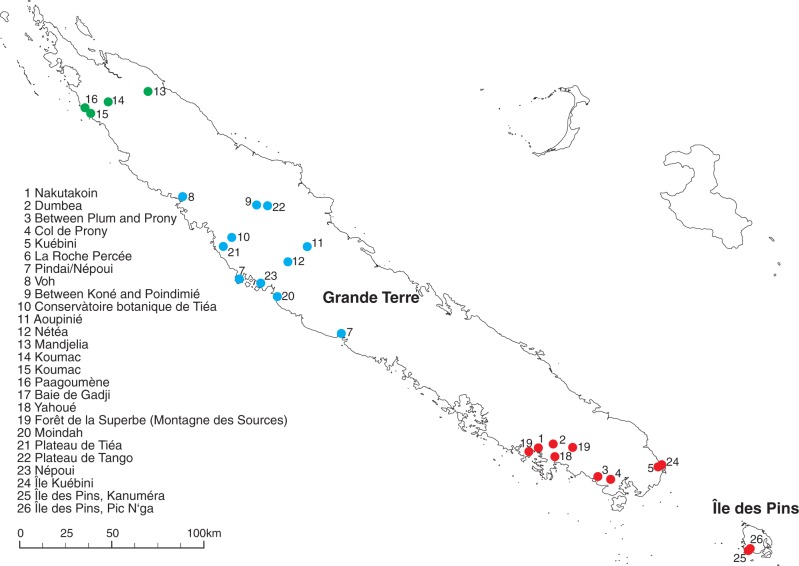
Map of New Caledonia indicating the 26 sampling localities for this study. Numbered dots indicate sampling sites (see also Table 1). Dots are coloured according to sampling region (north, green; middle, blue; south, red).

Leaf material from New Caledonian *Diospyros* species was collected on the main island, Grande Terre, and on one smaller island in the south, Île des Pins (Fig. 1). For the widespread species, at least two representative individuals were sequenced (Table 1). In total we included here 36 individuals of New Caledonian *Diospyros* species (corresponding to 21 identified and one unidentified species) as well as one individual of *Diospyros olen* (from New Caledonia, but not closely related to the other New Caledonian *Diospyros* species) and one individual of *Diospyros* *ferrea* from Thailand. *Diospyros olen* and *D. ferrea* were used as outgroups. Wherever possible, we used the exact same individuals for which Sanger sequence data are available from previous studies (Turner *et al.*, 2013*a*). However, because of poor DNA quality (unsuitable for NGS) or unavailability for some of the accessions, we had to include in this study another accession from the same population or locality.

**Table 1.** Table of accessions including all individuals used in this study. The identification numbers of sampling localities are given in Fig. 1. Voucher codes: JMXXXX: collection number J. Munzinger; Tree No. XXXXX: Tree of New Caledonian Plant Inventory and Permanent Plot Network (NC-PIPPN, Ibanez *et al*., 2014); KUFF, Herbarium of the Faculty of Forestry Kasertsat University Bangkok; MPU, Herbarium of the University of Montpellier; NOU, Herbarium of IRD Nouméa; P, Herbarium of the Natural History Museum Paris; WU, Herbarium of the University of Vienna

Taxon	Accession number	Sampling location	Voucher
*D. calciphila* F.White	BT313	25	MPU026746
*D. cherrieri* F.White	BT293	23	NOU079547
*D. erudita* F.White	BT280	21	WU062858
*D. ferrea* (Wild.) Bakh.	Eb045		Duangjai 106 (KUFF)
*D. flavocarpa* (Vieill. ex P.Parm.) F.White	BT130	9	MPU026741
	BT156	11	MPU026737
*D. glans* F.White	BT093	5	Turner *et al.* 093 (MPU)
*D. impolita* F.White	BT102	6	NOU019538
*D. inexplorata* F.White	BT308	24	NOU005818
*D. labillardierei* F.White	BT122	9	NOU052188
*D. minimifolia* F.White	BT135	10	NOU019556
	BT233	17	NOU019554
	BT263	20	NOU079549, WU062872
*D. olen* Hiern	BT001	1	NOU052191
*D. pancheri* Kosterm.	BT028	3	MPU026742
	BT031	3	MPU026742
*D. parviflora* (Schltr.) Bakh.	BT041	4	Turner *et al.* s.n. (NOU)
	BT090	5	NOU2519
	BT147	10	NOU052175
	BT187	13	NOU031409
	BT250	19	Tree no. 23109
	BT290	22	NOU079550
*D. perplexa* F.White	BT004	1	MPU026738
*D. pustulata* F.White	BT111	7	NOU019572
	BT140	10	NOU052177
	BT261	20	NOU079544
*D. revolutissima* F.White	BT120	8	NOU023189
	BT221	16	NOU084762
*D. tridentata* F.White	BT207	14	NOU052179
*D. trisulca* F.White	BT185	13	NOU031344
*D. umbrosa* F.White	BT176	12	JM6635 (NOU)
	BT247	19	NOU023234
*D. veillonii* F.White	BT227	17	NOU019582
*D. vieillardii* (Hiern) Kosterm.	BT025	2	Turner *et al.* s.n. (NOU)
	BT100	5	NOU006676
	BT215	15	NOU023242
*D. yahouensis* (Schltr.) Kosterm.	BT238	18	P00057340
*D.* sp. Pic N’ga	BT318	26	NOU054315

DNA was extracted from silica gel-dried leaf material using a modified sorbitol/high-salt cetyltrimethylammonium bromide (CTAB) method (Tel-Zur *et al.*, 1999). Extracts were purified using the NucleoSpin gDNA Clean-up kit (Marcherey-Nagel, Germany), according to the manufacturer’s protocol.

From each sample, 300 ng of DNA was sheared (in two cycles of 45 s with 30 s break between the two shearing runs) using an ultrasonicator (Bioruptor Pico, Diagenode, Belgium), targeting a mean fragment size of 400 bp. Library preparation was performed using the NEBNext Ultra DNA Library Prep Kit for Illumina (New England Biolabs, USA) according to the manufacturer’s protocol. All individuals were barcoded and pooled to reach an equal representation of each individual in the final libraries. In total, two libraries (containing 14 and 24 samples per library, respectively) were prepared for the 38 samples used. The two libraries were sequenced on an Illumina HiSeq as 100-bp paired-end reads at the VBCF (Vienna Biocenter Core Facilities, Vienna, Austria; http://www.vbcf.ac.at/facilities/next-generation-sequencing/). Demultiplexing of the raw data was performed, allowing for a maximum of one mismatch using the Picard BamIndexDecoder (included in the Picard Illumina2bam package; available from https://github.com/wtsi-npg/illumina2bam). The number and quality of raw reads obtained from each individual were evaluated with FastQC (Andrews, 2010).

### Assembling and annotating plastid genomes

Reads originating from the plastid genome (pt DNA) were filtered using a multistep and iterative in-house established pipeline. First, the individual raw files were imported into the CLC GENOMIC WORKBENCH v. 6.5 (Qiagen) and trimmed by quality at *P *<0·05, retaining reads of at least 30 bp. Then the reads of *D. ferrea* were mapped on the complete plastid genome of *Camellia sinensis* (Theaceae, Ericales, GenBank: KC143082.1). For this initial mapping of both coding and non-coding regions (the latter comprising introns and intergenic spacers), a mismatch cost of 2 and insertion and deletion cost of 3 were used, requiring at least 80 % of a read to be at least 90 % similar to the target for each successful mapping. With these settings, 403 980 reads of *D. ferrea* mapped to the *Camellia* plastome. We re-extracted the initial paired-end read data corresponding to these reads using FastQ.filter.pl (Rodriguez-R and Konstantinidis, 2016, available at https://github.com/lmrodriguezr/enveomics/) and have assembled them *de novo* in the CLC GENOMIC WORKBENCH, with automatic optimization of the word and bubble sizes and updating the contigs after mapping back the reads. We obtained three contigs, which have been concatenated by aligning them to the *C.* *sinensis* reference sequence manually in the program BioEdit v. 7.2.5 (Hall, 1999). From the coverage information and by comparison with the *C.* *sinensis* reference we were able to identify both inverted repeats and confirm their presence in the plastid genome of *Diospyros*. Both inverted repeats were reconstructed together and duplicated to represent a complete plastid genome. The base composition of the assembled contigs was extracted from the alignments using BioEdit v. 7.2.5 (Hall, 1999).

**Fig. 2.**
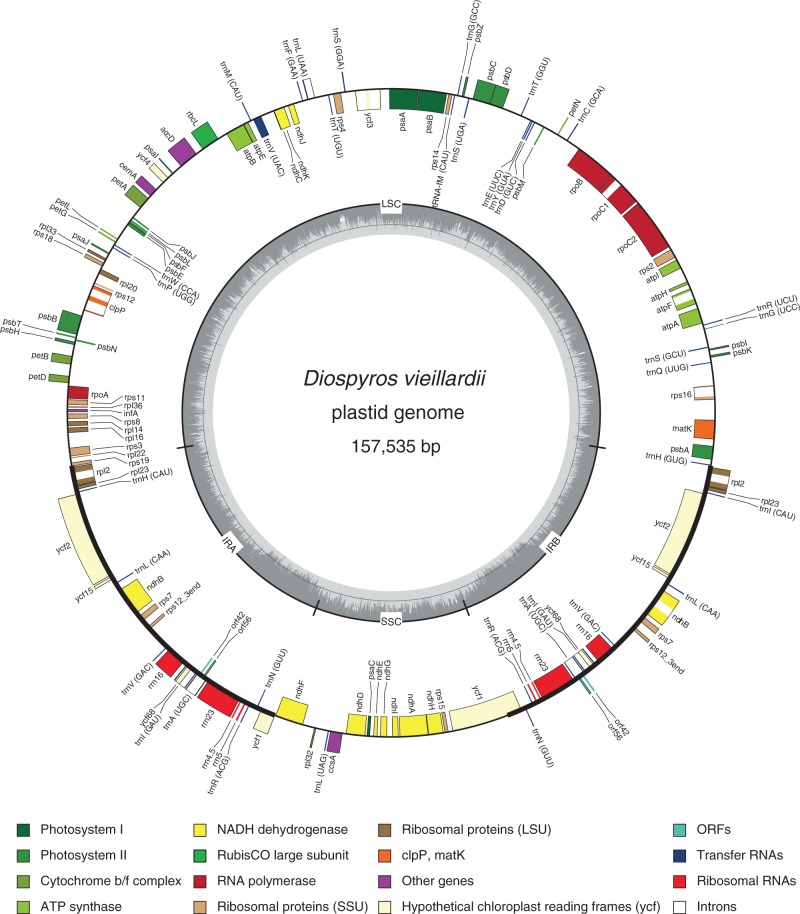
Graphic representation of the annotated plastid genome of *Diospyros vieillardii*.

The plastid genomes of the rest of the *Diospyros* species were obtained in a similar way, but the initial mapping step was performed on the assembled *D. ferrea* genome. Finally, annotation of coding regions was performed using DOGMA (Wyman *et al.*, 2004) using only the *D. vieillardii* BT025 plastid genome. The circular plastid genome map (Fig. 2) was visualized with OGDRAW (Lohse *et al.*, 2007).

Due to the difficulties with assembling the mitochondrial genome of plants as well as the low level of phylogenetic information in plant mtDNA (Malé *et al.*, 2014), we did not attempt to assemble the mitochondrial genome of *Diospyros*.

### Assembly of nrDNA repeats

Reads containing sequences from the nrDNA region were collected and assembled using the program MITObim v. 1.7 (Hahn *et al.*, 2013). As initial seed, we used previously generated Sanger sequences from the ITS region of *Diospyros* *vieillardii* BT025 (B. Turner, unpubl. res.). Characteristics of assemblies such as the number of reads assembled in each contig and coverage were inspected from the MITObim output files. Assembled sequences were aligned using Muscle v. 3.8 (Edgar, 2004). The alignment was manually inspected using the program BioEdit v. 7.2.5 (Hall, 1999). The beginning and end of each coding region were estimated by comparing the *Diospyros* alignment with annotated sequences of *Solanum lycopersicum* (GenBank: AY366529, AY552528). We also extracted the 5S nrDNA region using the procedure described above. As initial seed we used the 120-bp coding fragment of 5S nrDNA sequences of *Actinidia chinensis* (GenBank: AF394578).

### Phylogenetic analyses

For the phylogenetic analyses of the plastid sequence data, only one copy of the inverted repeat was included in the final alignment. For analyses of the nrDNA sequences, only the transcribed regions were used.

Parsimony analyses including bootstrapping were performed using PAUP* v. 4b10 (Swofford, 2003). They were run using a heuristic search with stepwise addition, random sequence addition (1000 replicates) and tree bisection–reconnection. Gaps were treated as missing data. To estimate clade support, bootstrapping with 1000 replicates was performed using the same settings as above (including 1000 random replicates per bootstrap replicate). Estimation of consistency index (CI) and retention index (RI) was done with PAUP. Likelihood analysis including bootstrapping was performed using RAxML v. 8.1.3 (Stamatakis, 2014). We used the Broyden–Fletcher–Goldfarb–Shanno (BFGS) method to optimize generalized time reversible (GTR) rate parameters, the gamma model of rate heterogeneity and 1000 rapid bootstrap inferences with a subsequent thorough maximum likelihood (ML) search. The results were visualized with FigTree 1·4 (available from http://tree.bio.ed.ac.uk/software/figtree/). We rooted the trees obtained with *D. olen* according to earlier results (Turner *et al.*, 2013*a*). 

To reduce the file size and speed up analyses in the combined analyses, *D. olen* and constant positions were removed using Mesquite v. 3.01 (Maddison and Maddison, 2014). In trees conducted from the combined data set *D. ferrea* was used as outgroup. Parsimony and likelihood analyses were performed as described for the individual data sets. In addition, we conducted a Bayesian analysis for the combined data set using BEAST v. 1.8.1 (Drummond *et al.*, 2012). The best evolutionary models for the two subsets (ptDNA and nrDNA) were evaluated using jModeltest v. 2.1.6 (Guindon and Gascuel, 2003; Darriba *et al.*, 2012). For the plastid partition, the transversional model with equal frequencies (TVMef; Posada, 2003) showed the best fit to the data, and for the nrDNA partition Tamura and Nei’s model (TrN; Tamura and Nei, 1993) with among-site rate variation modelled with a gamma distribution (TrN+Γ) showed the best fit. Base frequencies (uniform), substitution rates among bases (gamma shape 10) and alpha (gamma shape 10) were inferred by jModeltest for each data set. For flexibility, we used a relaxed uncorrelated log-normal clock model (Drummond *et al.*, 2006). As a speciation model, we used a Yule model because the investigated group is so young that we expected a low proportion of lineage extinction (Yule, 1925; Gernhard, 2008). For further details regarding the parameters used see Supplementary Data Fig. S1. Two independent Metropolis-coupled Markov chain Monte Carlo (MCMC) analyses, each with 20 million generations, were run, sampling each 1000th generation. The initial 10 % of trees obtained from each MCMC run were removed as burn-in; the remaining trees of both runs were used to calculate the maximum clade credibility tree. 

For directly comparing the results of the present study with previous ones (Turner *et al.*, 2013*a*) based on four plastid markers (*atpB*, *rbcL*, *trnK*-*matK*, *trnS*-*trnG*; 5979 bp) and two low-copy nuclear markers (*ncpGS*, 717 bp; *PHYA*, 1189 bp), a subset of those data corresponding to the individuals included in this study was analysed in the same way as described above for the Bayesian analysis. In most cases, to represent each species we used either the same accession or individuals from the same population. In the few cases where no data from individuals of the same population were available, then the geographically closest individual assigned to the same species was used. Due to these differences, results of the previous studies are not strictly comparable to those produced here, but we have compared them nonetheless due to their overall similarity.

A general pattern of geographical clustering was visually observed in the resulting trees, in particular in the plastid data. Based on the geographical coordinates of samples and the distance matrix of pairwise uncorrected *P*-values (calculated with SplitsTree), we tested the significance of geographical clustering of the samples in the trees using a Mantel test. We estimated the correlation of geographical and genetic discontinuities in the data by performing this analysis on the plastid and the combined data sets with Isolation by Distance web service (IBDWS) (Jensen *et al.*, 2005).

To investigate the relationships between populations we constructed a neighbour network based on the plastid markers, using SplitsTree4 v. 4.13.1 (Hudson and Bryant, 2006) and uncorrected *P* distance estimates. Based on the results from the Mantel test [Table 2 and RADseq results (Paun *et al.*, 2016)], we excluded samples of *D. olen*, *D. ferrea*, *D. vieillardii*, *D. umbrosa*, *D. flavocarpa*, *D. cherrieri* and *D. veillonii* from this analysis to get a clearer picture of the relationships within the recently and rapidly radiated species group.

**Table 2.** The extent of geographical clustering in the data, as evidenced with Mantel tests performed on IBDWS

Dataset	Mantel’s *r*	Significance
Plastid data		
Including all individuals, except the outgroups *D. olen* and *D. ferrea*	0·103	*P* = 0·09
Excluding *D. olen*, *D. ferrea* and all *D. vieillardii*	0·242	*P* < 0·001
Excluding *D. olen*, *D. ferrea*, *D. vieillardii*, *D. umbrosa* and *D. flavocarpa*	0·383	*P* < 0·001
Excluding *D. olen*, *D. ferrea*, *D. vieillardii*, *D. umbrosa*, *D. flavocarpa*, *D. cherrieri* and *D. veillonii*	0·427	*P* < 0·001
Combined		
Including all individuals except the outgroups *D. olen* and *D. ferrea*	0·079	*P* = 0·10
Excluding *D. olen*, *D. ferrea* and all *D. vieillardii*	0·121	*P* < 0·05
Excluding *D. olen*, *D. ferrea*, *D. vieillardii*, *D. umbrosa* and *D. flavocarpa*	0·302	*P* < 0·001
Excluding *D. olen*, *D. ferrea*, *D. vieillardii*, *D. umbrosa*, *D. flavocarpa*, *D. cherrieri* and *D. veillonii*	0·374	*P* < 0·001

**Table 3.** Details of samples used here for sequencing of whole plastid genomes and nrDNA

	Raw reads	Plastid genome			Nuclear ribosomal DNA		
		Percentage of reads mapping	Coverage (×)	GC (%)	Percentage of reads mapping	Coverage (×)	GC (%)
*D. calciphila* BT313	19549968	1·34	162	37·37	0·17	444	59·24
*D. cherrieri* BT293	15164658	3·30	309	37·36	0·11	229	58·52
*D. erudita* BT280	12612118	3·03	236	37·36	0·15	246	59·36
*D. ferrea* Eb045	9943646	4·81	300	37·34	0·51	559	58·46
*D. flavocarpa* BT130	10129514	0·99	62	37·36	0·29	331	57·07
*D. flavocarpa* BT156	15656792	0·53	51	37·37	0·10	219	58·53
*D. glans* BT093	14436316	0·91	81	37·36	0·19	386	58·48
*D. impolita* BT102	18636398	1·14	131	37·36	0·08	209	59·26
*D. inexplorata* BT308	16201056	1·06	106	37·36	0·24	511	59·65
*D. labillardierei* BT122	26574012	0·95	156	37·36	0·15	538	58·6
*D. minimifolia* BT135	26685314	1·19	199	37·36	0·09	314	58·82
*D. minimifolia* BT233	25526086	0·84	134	37·35	0·13	384	58·24
*D. minimifolia* BT263	16154630	1·95	198	37·35	0·29	616	59·14
*D. olen* BT001	24966688	1·51	236	37·44	0·38	1084	58·32
*D. pancheri* BT028	27590124	0·80	136	37·35	0·09	316	59·38
*D. pancheri* BT031	29453086	0·71	130	37·36	0·09	361	58·98
*D. parviflora* BT041	27178316	0·83	140	37·36	0·09	356	58·54
*D. parviflora* BT090	25432978	0·40	63	37·37	0·19	656	59·34
*D. parviflora* BT147	17887304	0·65	71	37·36	0·28	690	58·69
*D. parviflora* BT187	26648984	0·45	74	37·36	0·21	690	56·6
*D. parviflora* BT250	17588828	0·43	47	37·36	0·37	877	59·02
*D. parviflora* BT290	19187356	0·39	47	37·36	0·28	737	59·08
*D. perplexa* BT004	18085506	0·74	82	37·36	0·30	595	57·43
*D. pustulata* BT111	15958418	2·46	242	37·36	0·28	467	57·69
*D. pustulata* BT140	17029506	1·80	188	37·36	0·26	580	59·52
*D. pustulata* BT261	15585090	1·19	114	37·36	0·37	745	59·94
*D. revolutissima* BT221	20867576	1·50	193	37·37	0·20	551	57·98
*D. revolutissima* BT120	17111770	1·46	154	37·35	0·21	466	59·16
*D. tridentata* BT207	14190292	0·89	77	37·35	0·21	356	57·79
*D. trisulca* BT185	17297816	0·75	80	37·36	0·29	710	58·67
*D. umbrosa* BT176	18856642	0·85	98	37·38	0·48	632	55·92
*D. umbrosa* BT247	28845756	0·59	104	37·36	0·10	385	59·19
*D. veillonii* BT227	26594158	1·80	297	37·36	0·30	1056	58·65
*D. vieillardii* BT025	26595776	0·91	152	37·34	0·38	1160	58·29
*D. vieillardii* BT100	22649344	2·10	297	37·33	0·11	327	58·52
*D. vieillardii* BT215	27710030	0·80	138	37·32	0·14	501	58·94
*D. yahouensis* BT238	29242716	0·80	144	37·36	0·15	578	59·68
*D.* sp. Pic N’ga BT318	18515350	4·63	531	37·36	0·21	500	59·37

## RESULTS

After the demultiplexing step, the number of raw Illumina sequences ranged from 10 to 29 million paired-end reads per individual. Details of sample characteristics are given in Table 3.

### Plastid genomes

We obtained between 75 262 (47× average coverage, for *D. parviflora* BT250) and 857 039 (531× coverage, for *D.* sp. Pic N’ga BT318) pairs of reads per individual that mapped to the plastid genome (for details see Table 3). The GC content of the plastid genomes of *Diospyros* (∼37 %) is similar to those of many other angiosperms [average ∼37 %; e.g. *Ardisia* (Ku *et al.*, 2013); *Camellia* (Yang *et al.*, 2013); *Potentilla* (Ferrarini *et al.*, 2013); *Musa* (Martin *et al.*, 2013); Zingiberales (Barrett *et al.*, 2014)].

The size (∼ 157 kb) and gene order of the plastid genome of *D.* *vieillardii* (Fig. 2) is similar to that of *C.* *sinensis* (GenBank: KC143082.1). This plastid genome is the first fully sequenced plastid genome of Ebenaceae reported in the literature.

The plastid matrix of *Diospyros* used for phylogenetic analyses (including only one of the inverted repeats) included 133 210 characters, of which 1295 variable positions were parsimony-uninformative and 384 (0·3 %) were potentially parsimony-informative. Phylogenetic reconstruction using parsimony produced 1127 equally parsimonious trees (results not shown) with a CI of 0·93 and RI of 0·87. Phylogenetic relationships between the species were in many cases not well supported, and in several cases individuals of the same species failed to form well-supported clusters. Similar results were obtained using maximum likelihood (Supplementary Data Fig. S2).

### Nuclear ribosomal DNA

Between 0·08 % (*D. impolita* BT102) and 0·51 % (*D. ferrea* Eb045) of the total reads pertained to the nrDNA region (for details see Table 3). The contigs obtained had average coverages ranging from 209× (for *D. impolita* BT102) to 1159× (for *D. vieillardii* BT025). The NTS of the intergenic spacer was difficult to align and therefore excluded from further analyses. The aligned nrDNA matrix of *Diospyros* included 7233 characters, of which 368 variable positions were parsimony-uninformative and 141 (1·9 %) were potentially parsimony-informative. The parsimony phylogenetic reconstruction with only the nrDNA sequences produced 84 equally parsimonious trees (results not shown) with a CI of 0·66 and RI of 0·53. Phylogenetic relationships generally disagreed with results obtained from other markers, but these incongruent relationships are all weakly supported (results not shown). Several species failed to form unique groups. Similar results were obtained using maximum likelihood (Supplementary Data Fig. S3).

The assembled 5S nrDNA region containing coding and non-coding parts was short (less than 500 bp) and therefore did not contain many informative characters. Phylogenetic trees based on this fragment were poorly resolved, and therefore the trees are not presented or discussed further. The 5S nrDNA region was also not included in the combined analysis.

### Analyses of the combined data set

In addition to the individual analyses of the two data sets, we also combined them to determine whether this approach provides better resolution/support.

**Fig. 3.**
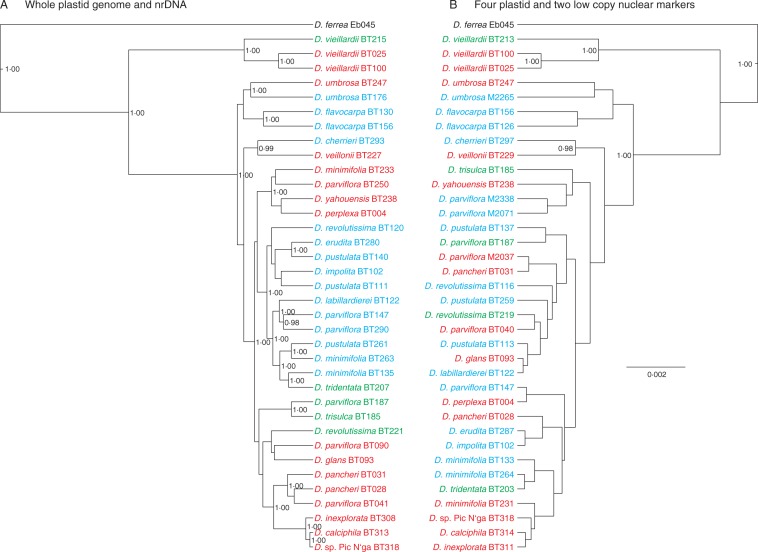
Phylogenetic trees resulting from the Bayesian analysis of the (A) combined data set of the present study (variable nucleotide positions in the whole plastid genome and nrDNA) and (B) combined data set of a previous study including four plastid and two low-copy nuclear markers (Turner *et al.*, 2013*a*). The numbers indicate posterior probabilities >90. Trees are scaled to the same branch length. Samples are coloured according to sampling region (see Fig. 1).

The combined matrix (i.e. reduced to variable positions as explained in Materials and methods) included 1136 characters, of which 437 were potentially parsimony-informative. Phylogenetic reconstruction using parsimony produced a single most parsimonious tree of 1580 steps (Supplementary Data Fig. S4) with a CI of 0·73 and RI of 0·52. Phylogenetic relationships depicted in the combined analysis were better resolved than in the trees obtained from the individual analyses. A comparable pattern was found in the Bayesian analysis of the combined data set (Fig. 3). All three individuals of *D. vieillardii* clustered together and were sister to the rest of the New Caledonian endemic species group. Individuals from *D. flavocarpa* and *D. umbrosa* formed unique groups and were sister to the rest of the remaining accessions, among which *D. cherrieri* and *D. veillonii* were sister to the rest. The results from the combined analysis were also in agreement with earlier results based on plastid and nuclear markers (Turner *et al.*, 2013*a*).

There is a trend of geographical clustering visible in the Bayesian tree (Fig. 3) and in the neighbour network (Supplementary Data Fig. S5). The neighbour network (Fig. S5) clearly shows that individuals and populations from the south and the middle of New Caledonia clustered according to their sampling region. The Mantel test (Table 2) confirmed a significant geographical clustering of the genetic information (Fig. 3), in particular in the plastid data across the crown group.

## DISCUSSION

Previous standard approaches to phylogenetic analysis of *Diospyros* species including samples from New Caledonia used nine (Duangjai *et al.*, 2009) and four (Turner *et al.*, 2013*a*) plastid markers (alignment length >8 and 6·4 kb, respectively). They demonstrated low levels of sequence divergence among these species, indicating a fairly recent and rapid radiation. Inclusion of low-copy nuclear markers, which have been shown to be highly informative and useful for resolving phylogenetic relationships at lower taxonomic levels in some taxa [e.g. *Paeonia* (Tank and Sang, 2001) and *Passiflora* (Yockteng and Nadot, 2004)], only partly improved resolution among the New Caledonian *Diospyros* species (Turner *et al.*, 2013*a*). Similar results have been observed in two genera of Cunoniaceae from New Caledonia [*Spiraeanthemum* (Pillon *et al.*, 2009*a*) and *Codia* (Pillon *et al.*, 2009*b*)].

AFLP markers are typically used at low taxonomic level in closely related species and population analyses (e.g. Despré *et al.*, 2003; Tremetsberger *et al.*, 2006; Gaudeul *et al.*, 2012). In the case of the New Caledonian *Diospyros* species, we used AFLPs to evaluate species limits (Turner *et al*., 2013*b*), and in most cases we found congruence with the species concepts of White (1993). However, the AFLP approach was not useful in resolving phylogenetic relationships among these species (Turner *et al*., 2013*b*) because there were few of the individual AFLP fragments shared by two or more species. It would appear from the AFLP results that fragmentation of an original widespread population occurred in many regions of New Caledonia more or less simultaneously, resulting in genetically distinct populations that correspond to the morphologically based species delimitations of White (1993) without leaving much evidence for interspecific relationships.

The phylogenetic tree obtained here based on the whole plastid genome (Fig. S2) is similar to the phylogenetic tree previously based on four plastid markers (Turner *et al.*, 2013*a*). The combined tree of this study (Fig. 3A) is similar both in resolution and structure to the phylogenetic tree based on four plastid and two low-copy nuclear markers (Fig. 3B). Although not always represented by the same individuals, general relationships of species are in agreement.

The nuclear ribosomal region included a higher percentage of parsimony-informative characters (1·9 versus 0·3 %) than the plastid DNA, which is in agreement with findings in other plant groups (Hamby and Zimmer, 1992; Doyle, 1993; Malé *et al.*, 2014). Despite this variability, nrDNA was still too short to contain enough variation (141 versus 384 informative sites) and failed to resolve phylogenetic relationships among the New Caledonian *Diospyros* species (Fig. S3).

Our results clearly show that for this group of species, in which standard plastid and nuclear markers were not helpful for resolving the phylogenetic relationships, using the whole plastid genome does not greatly increase resolution and support. There are only a few other studies available in which whole plastid genomes have been used to resolve phylogenetic relationships at the intraspecific level among closely related species. Some studies [e.g. in Chrysobalanaceae (Malé *et al.*, 2014) and eucalypts (Myrtaceae) (Bayly *et al.*, 2013)] have revealed that this approach can be useful for resolving phylogenetic relationships among genera, but they failed to resolve phylogenetic relationships among closely related species and to group together individuals of the same taxonomic species. In cases of recently radiating species groups, in particular following an extreme bottleneck associated with a long-distance dispersal event such as the arrival of *Diospyros* in New Caledonia, plastid genomes appear to be insufficient for inference of phylogenetic relationships. The basis of the rapid radiation of *Diospyros* in New Caledonia is not yet clear, but it has been speculated that it is an adaptive origin associated with different soil types (Paun *et al.*, 2016), as recently shown for the genus *Geissois* in Cunoniaceae (Pillon *et al.*, 2014).

The individuals of *D. vieillardii*, *D. umbrosa*, *D. flavocarpa*, *D. cherrieri* and *D. veillonii* form a minimally isolated group in the combined analyses (Fig. 3). These species form clusters that are successively sister to the rest of the taxa, which are well supported collectively but form a highly unresolved central cluster. This unresolved central cluster is less than 6 million years old (Paun *et al.*, 2016) and could be the result of two lineages that developed in isolation and then subsequently colonized some of the same habitats. This too could be the result of simultaneous parallel divergence in different parts of New Caledonia combined with effects of local and more recent hybridization (as was indicated in the AFLP study; Turner *et al*., 2013*b*). Retention of ancient polymorphisms present in the original colonizing population, which probably also underwent a severe bottleneck, could not produce such a geographically structured pattern.

Comparisons of the phylogenetic tree based on plastid sequences (Fig. S2) with the tree derived from RAD data [Paun *et al.*, 2016 (Supplementary Data Fig. S6)] showed several clusters of individuals (*D. trisulca* and *D. parviflora* from L13, *D. pustulata* and *D. minimifolia* from L20, *D. pancheri* and *D. parviflora* from L3 and 4; Table 1, Fig. 1) that occur with high statistical support in the plastid results, but are not present in the nuclear tree. These clusters consist of individuals found in the same or very nearby locations, which could indicate introgressive hybridization and transfer of plastid genomes as a relevant phenomenon (Naciri and Linder, 2015, and references therein). Geographical rather than taxonomic clustering was observed for all populations of *D. minimifolia* and *D. parviflora* that also failed to form unique groups in nuclear results [AFLP (Turner *et al.*, 2013*b*) RAD (Paun *et al.*, 2016)]. Phenomena like introgressive hybridization and transfer of plastid genomes could also explain the geographical clustering of individuals observed in the plastid data set (Figs S2 and S5; Table 2), whereas in nuclear-based data sets [AFLP (Turner *et al.*, 2013*b*), RAD (Paun *et al.*, 2016)] no such geographical clustering was observed. Similar geographical clustering for plastid results has previously been reported in other plant groups [e.g. *Nothofagus* (Acosta and Premoli, 2010)].

Although there are more than 140 kb of DNA sequence included in this study, it effectively corresponds to only two markers (plastid genome and rDNA region). As not all genes evolve in the same mode and at the same tempo, phylogenies based on different genes might show different phylogenetic relationships (Heled and Drummond, 2010). It is therefore important to use many phylogenetic markers for reconstruction of relationships to overcome the limitations of individual genes and produce results as close as possible to the real species tree. Phylogenetic trees based on plastid data should always be viewed as gene trees because of the typical evolutionary pathways of these organelles (Naciri and Linder, 2015). The trees presented here may hence show the evolutionary history of the particular region investigated, and may differ from the species trees. In the case of the New Caledonian *Diospyros* species, we consider the SNP data derived from RAD (Paun *et al.*, 2016) as the best approximation of the species tree, because it involves nearly 8500 independent markers from the nuclear genome.

## Conclusions

Although New Caledonian *Diospyros* are morphologically and ecologically diverse, they show little genetic divergence. For these rapidly radiating *Diospyros* species, in which standard plastid and nuclear markers were not helpful for resolving the phylogenetic relationships, using the whole plastid genome does not greatly increase resolution and support. Plastid markers grouped accessions according to geographical provenance, which could result from local transfer of plastid genomes due to hybridization and introgression following secondary contact.

We are now conducting additional nuclear genome studies (both coding and non-coding regions) to determine whether other approaches could help us determine the potential adaptive nature of this radiation, which has thus far defeated our attempts using standard and next-generation methods.

## SUPPLEMENTARY DATA

Supplementary data are available online at www.aob.oxfordjournals.org and consist of the following. Figure S1: BEAST input file for the Bayesian analysis of the combined data set including all settings and priors of both data sets. Figure S2: maximum likelihood tree based on plastid sequences. Figure S3: maximum likelihood tree based on nrDNA sequences. Figure S4: single most parsimonious tree based on the combined data matrix. Figure S5: neighbour network based on the plastid data set. Individuals are coloured according to their sampling region. Figure S6: maximum likelihood tree based on RAD SNP data.

Supplementary Data
