# Supplementary material for: Sequencing of whole plastid genomes and nuclear ribosomal DNA of Diospyros species (Ebenaceae) endemic to New Caledonia: many species, little divergence
Source: Ann Bot. 2016 Apr 20;117(7):1175–85. doi: 10.1093/aob/mcw060 (PMC4904177; doi:10.1093/aob/mcw060)
Supplement: Supplementary Data [file supp_117_7_1175__index.html]

Sequencing of whole plastid genomes and nuclear ribosomal DNA of Diospyros species (Ebenaceae) endemic to New Caledonia: many species, little divergence — Supplementary Data 

# Sequencing of whole plastid genomes and nuclear ribosomal DNA of *Diospyros* species (Ebenaceae) endemic to New Caledonia: many species, little divergence

## Supplementary Data

files

- Supplementary Data - zip file
